# Multivitamins in Adult Medical Practice: Evidence, Risks, and Pragmatic Prescribing

**DOI:** 10.7759/cureus.101985

**Published:** 2026-01-21

**Authors:** Pedro M Neves, Sofia Almeida, Vital Domingues

**Affiliations:** 1 Internal Medicine, Unidade Local de Saúde de Santo António, Porto, PRT; 2 Internal Medicine, Centro Hospitalar Universitário de Santo António, Porto, PRT

**Keywords:** alcohol use disorder, all-cause mortality, bariatric surgery, cancer incidence and mortality, cardiovascular events, internal medicine, multivitamin-mineral supplements

## Abstract

Use of multivitamin-mineral supplements is common and often occurs outside clinical care. Evidence for broad preventive benefit remains uncertain. We synthesize validated indications, clinical benefits, and harms, and offer a pragmatic prescribing approach for internists.

Narrative review of guidelines, randomized trials, and meta-analyses indexed in major databases (2000-August 2025). Priority was given to high-level evidence on hard outcomes (all-cause mortality, cardiovascular events, cancer incidence/mortality, cognition) and to conditions frequently managed in Internal Medicine (malabsorption, bariatric surgery, alcohol use disorder, aging, restrictive diets). This is not a systematic review.

Multivitamins are justified for documented deficiencies or high-risk states, including malabsorption, post-bariatric surgery, chronic alcohol use, pregnancy/lactation, and frailty. In generally nourished adults, multivitamins do not reduce cardiovascular events or mortality, and evidence for cancer prevention is marginal without a mortality impact. Recent data suggest small cognitive benefits in older adults. Risks include hypervitaminosis A/D/E, clinically relevant drug-nutrient interactions, masking of specific deficiencies, behavioral displacement of diet-first strategies, and unnecessary cost.

Outside of deficiency or clearly increased need, routine multivitamin prescribing provides little clinical gain and exposes patients to avoidable harms. Internists should screen, test, and treat specifically, prefer targeted nutrients, monitor safety, and educate patients against overreliance on supplements.

## Introduction and background

Across high-income countries, multivitamin-mineral (MVM) use is part of daily life for many adults. In large surveys from North America and Europe, up to half of adults report regular supplement use, often without a confirmed deficiency and outside medical supervision [1-3]. For internists, who manage multimorbidity and polypharmacy, the idea that “natural equals safe” is attractive yet misleading.

Despite popularity, the clinical payoff is modest. Meta-analyses and guidance documents have repeatedly shown limited or absent benefit of routine MVM use in primary prevention, with small and inconsistent signals in cancer incidence and more nuanced results in cognition [4-6]. Meanwhile, fat-soluble vitamin toxicity, drug-nutrient interactions, and diagnostic masking are daily realities in Internal Medicine. This review distills when MVMs help, when they do not, and how to decide with patients in front of us.

We conducted a pragmatic literature search (2000-August 2025) of MEDLINE, Embase, and the Cochrane Library using combinations of multivitamin, micronutrient, supplement, and deficiency, alongside outcome terms (mortality, cardiovascular, cancer, cognition). We prioritized randomized trials, meta-analyses, and clinical guidelines relevant to adult Internal Medicine. Reference lists of key papers were screened. This is a narrative synthesis aimed at clinical decision-making.

## Review

Validated clinical indications

Multivitamins, or preferably targeted single-nutrient therapy, are appropriate when a deficiency is documented or highly probable.

Vitamin B12 supplementation is indicated in older adults, strict vegetarians or vegans, and in patients with pernicious anemia, atrophic gastritis, or long-term proton pump inhibitor use [7]. Vitamin D deficiency is common among institutionalized individuals, those with obesity, limited sun exposure, or chronic kidney disease [8]. Iron and folate supplementation are warranted in women of reproductive age, during pregnancy, in chronic blood loss, or in the presence of malabsorption [9].

In malabsorption syndromes and after bariatric surgery, such as in celiac disease, Crohn’s disease with ileal involvement, and pancreatic insufficiency, sustained multimicronutrient repletion (including vitamins B12 and D, iron, zinc, and fat-soluble vitamins) with regular monitoring is recommended [10].

In alcohol use disorder, early combined repletion of thiamine, vitamin B6, folate, vitamin B12, and vitamins A and D is standard practice, particularly during withdrawal and refeeding [11].

Physiological states such as pregnancy and lactation justify supplementation with folate, iron, vitamin D, and iodine in accordance with national guidelines; adolescence and frailty may also require targeted nutritional support based on clinical assessment [9].

Restrictive diets and eating disorders, including strict veganism or severely limited intake, should first be addressed through dietary counseling, with targeted supplementation of vitamin B12, vitamin D, iron, calcium, and zinc as indicated [12].

Do multivitamins improve outcomes?

Evidence does not support the routine use of MVM supplements for the prevention of myocardial infarction, stroke, or cardiovascular mortality in generally well-nourished adults [13]. The U.S. Preventive Services Task Force advises against the use of beta carotene or vitamin E for cardiovascular prevention and concludes that evidence is insufficient to recommend MVMs for primary prevention [14].

Regarding cancer, the Physicians’ Health Study II found that daily MVM supplementation led to a modest 8% reduction in total cancer incidence among male physicians over an 11-year follow-up, without associated mortality benefit and with diminishing effect across tumor types [15]. This finding has not led to population-wide recommendations.

In cognition, the COSMOS-Mind trial reported small improvements in global cognition and episodic memory over three years among older adults taking daily MVMs, with stronger effects observed in those with prior cardiovascular disease. The effect size is modest and should be interpreted as targeted rather than generalizable [16].

For respiratory infections, including COVID-19, correcting true micronutrient deficiencies (such as vitamin D or zinc) is reasonable in vulnerable groups, but universal supplementation has not demonstrated reduced infection risk or improved outcomes in the general population [17].

Overall, outside of clear deficiency or elevated risk, routine MVM use does not alter major clinical outcomes. The most credible current signal is a modest cognitive benefit in selected older adults.

Risks and limitations of prolonged use

Chronic excessive intake of fat-soluble vitamins can lead to clinically significant toxicity. Vitamin A excess is associated with hepatic injury, headache, dry skin, and bone fragility [18]. High doses of vitamin D without appropriate monitoring can cause hypercalcemia, arrhythmias, and nephrolithiasis [19]. Meta-analyses suggest that high-dose vitamin E supplementation may slightly increase all-cause mortality [20].

Several important drug-nutrient interactions should also be considered. Vitamin K reduces the anticoagulant effect of warfarin. Calcium, magnesium, and zinc can chelate fluoroquinolones and tetracyclines, lowering antibiotic bioavailability. Calcium also decreases the absorption of levothyroxine, and high-dose vitamin C may alter salicylate excretion. Concrete prescribing and monitoring guidance for these interactions is summarized in Table 1.

**Table 1:** Clinically relevant drug-nutrient interactions INR: international normalized ratio

Supplement	Interacting drug	Clinical effect	Recommendation
Vitamin K	Warfarin	Reduced anticoagulant effect	Monitor INR; keep intake stable
Calcium	Levothyroxine	Reduced absorption	Separate by ≥4 hours
Magnesium/zinc/calcium	Fluoroquinolones, tetracyclines	Reduced antibiotic bioavailability	Separate by ≥2 hours
Vitamin C (high dose)	Salicylates	Increased renal excretion	Monitor efficacy/toxicity

Folate supplementation can correct macrocytosis while allowing neurological damage from unrecognized vitamin B12 deficiency to progress.

Finally, MVM supplements can create a false sense of security, leading patients to neglect dietary quality, physical activity, sleep, and medication adherence. At a population level, this behavioral displacement contributes to considerable expenditure with minimal clinical yield outside clearly defined indications [21].

The evidence supporting these findings is summarized in Tables 1-4.

**Table 2:** Validated clinical indications for supplementation (condensed)

Clinical situation	Likely deficits	Recommendation
Documented deficiency	B12, 25‑OH‑D, folate, iron	Yes; targeted and monitored
Malabsorption or post‑bariatric	A, D, E, K, B12, folate, Ca, Mg, Zn	Yes; individualized, long‑term
Alcohol use disorder	Thiamine, B6, B12, folate, A, D	Yes; early, combined
Pregnancy/lactation	Folate, iron, vitamin D, iodine, calcium	Yes; per guideline
Frailty/aging	D, B12, zinc	Yes; screen and recheck
Strict veganism/restriction	B12, D, iron, calcium, zinc	Diet‑first plus targeted supplements

**Table 3:** Evidence summary by clinical domain CVD: cardiovascular; MI: myocardial infarction; MVM: multivitamin

Domain	Population	Intervention	Outcome	Conclusion
Cardiovascular	Generally healthy adults	MVM/vitamin D/calcium/vitamin C	MI, stroke, CVD mortality	No benefit
Cancer	Men ≥50 years	MVM vs placebo	Total cancer; mortality	Small ↓ in incidence; no mortality effect
Cognition	Older adults	MVM vs placebo	Global cognition, episodic memory	Modest benefit (subgroup‑driven)
Immunity/COVID‑19	Adults/older adults	MVM + D + C + zinc	Infection risk/severity	No population effect; benefit when correcting deficits

**Table 4:** Specific indications for calcium and vitamin C

Clinical scenario	Micronutrient	Rationale	Recommendation
Osteoporosis prevention/treatment	Calcium with vitamin D	Bone mineral density	Yes; co‑administer with D, avoid excess
Hypoparathyroidism	Calcium	Chronic hypocalcemia	Yes; biochemical monitoring
Chronic glucocorticoid therapy	Calcium + vitamin D	Drug‑induced bone loss	Yes; preventive
Dairy‑free diets/veganism	Calcium	Low dietary intake	Consider after dietary assessment
Oral iron therapy	Vitamin C	Improves non‑heme iron absorption	Consider co‑administration
Scurvy (rare)	Vitamin C	Replacement therapy	Targeted treatment only

## Conclusions

Multivitamins are not harmless placeholders. In Internal Medicine, they have a clear role when deficiencies are documented or the risk is high. Elsewhere, benefits are scant, and risks and costs are real. The best approach is targeted, time-limited, and monitored supplementation within a diet-first framework. Internists are well placed to screen, test, deprescribe when appropriate, and educate patients with clear, balanced messages.
